# Periodontal Disease and Oral Squamous Cell Carcinoma: A Systematic Review and Meta-Analysis of Risk and Survival Outcomes

**DOI:** 10.3390/jcm15083161

**Published:** 2026-04-21

**Authors:** Gabriela Guadalupe Zambrano Manzaba, Luis Chauca-Bajaña, Carlos Andrés Guim Martínez, Sara Isabel Marcalupo Llerena, Andrea Ordoñez Balladares, Byron Velasquez Ron, Carlos Enrique Cuevas-Suárez, Abigailt Flores-Ledesma, Veronica Natalia Maroto Hidalgo, Gina Fernanda Vásquez Armas

**Affiliations:** 1 School of Dentistry, Universidad Católica de Santiago de Guayaquil (UCSG), Guayaquil 090101, Ecuador; 2 College Dentistry, University of Guayaquil, Guayaquil 090101, Ecuador; luis.chaucab@ug.edu.ec (L.C.-B.);; 3 College Dentistry, Universidad Bolivariana del Ecuador, Durán 092406, Ecuador; 4 Carrera de Odontología, Department Prosthesis Research, Universidad de Las Américas (UDLA), Quito 170102, Ecuador; byron.velasquez@udla.edu.ec; 5 Dental Materials Laboratory, Academic Area of Dentistry, Autonomous University of Hidalgo State, San Agustín Tlaxiaca 42160, Mexico; cecuevas@uaeh.edu.mx; 6 Dental Materials and Biomaterials Laboratory, Faculty of Stomatology, Meritorious Autonomous University of Puebla, Puebla 72590, Mexico

**Keywords:** periodontal diseases, mouth neoplasms, carcinoma, squamous cell, *Porphyromonas gingivalis*, survival analysis

## Abstract

**Background/Objectives:** Oral squamous cell carcinoma (OSCC) accounts for over 90% of oral malignancies and remains associated with substantial global morbidity and mortality. Although tobacco and alcohol are established risk factors, they do not fully explain OSCC incidence, highlighting the need to explore additional contributors such as chronic inflammatory conditions. Periodontal disease, characterized by persistent inflammation and microbial dysbiosis, has emerged as a plausible factor in oral carcinogenesis and tumor progression. To systematically evaluate the association between periodontal disease and the risk of developing OSCC, and to assess the prognostic impact of periodontal disease–related factors, particularly intratumoral *Porphyromonas gingivalis*, on survival outcomes in patients with OSCC. **Methods:** A systematic review and meta-analysis were conducted in accordance with PRISMA guidelines and prospectively registered in PROSPERO (CRD420261296479). Comprehensive searches were performed in PubMed, EMBASE, Web of Science, Scopus, the Cochrane Library, ClinicalTrials.gov, and World Health Organization regional databases. Case–control studies evaluating OSCC risk and cohort studies assessing survival outcomes were included. Random-effects meta-analyses using inverse-variance models were applied. Heterogeneity was assessed using the I^2^ statistic, and robustness was evaluated through Hartung–Knapp adjustment, leave-one-out sensitivity analyses, and Trial Sequential Analysis. **Results:** Five case–control studies were included in the etiological analysis. Periodontal disease was significantly associated with an increased risk of OSCC (pooled OR = 3.17; 95% CI: 1.94–5.21), with moderate heterogeneity (I^2^ = 58.7%). Two cohort studies were included in the prognostic analysis. High intratumoral expression of *P. gingivalis* was significantly associated with poorer overall survival (pooled HR = 2.15; 95% CI: 1.33–3.47), with no detected heterogeneity (I^2^ = 0%). **Conclusions:** Periodontal disease is strongly associated with an increased risk of OSCC, and intratumoral *P. gingivalis* appears to be an adverse prognostic marker. These findings underscore the relevance of periodontal inflammation and microbial factors across the OSCC continuum, from carcinogenesis to clinical outcomes, and support their consideration as potential targets for risk stratification and prevention strategies.

## 1. Introduction

Oral squamous cell carcinoma (OSCC) represents more than 90% of malignancies arising in the oral cavity and remains a major global health burden due to its high morbidity, substantial mortality, and frequent diagnosis at advanced stages. Despite advances in surgical techniques, radiotherapy, and systemic therapies, overall survival has shown only modest improvement over recent decades, underscoring the importance of identifying modifiable risk factors and prognostic determinants that could inform prevention strategies and improve patient outcomes [1,2,3].

Tobacco use and alcohol consumption are well-established etiological factors for OSCC; however, these exposures do not fully explain the observed incidence patterns, particularly among individuals without traditional risk factors [4,5]. This gap has prompted growing interest in the role of chronic inflammatory conditions and the oral microbiome in oral carcinogenesis. Periodontal disease, a highly prevalent chronic inflammatory disorder characterized by persistent microbial dysbiosis and progressive destruction of the tooth-supporting tissues, has emerged as a plausible contributor to oral cancer development [6,7,8].

From a biological perspective, periodontitis creates a sustained pro-inflammatory microenvironment marked by elevated levels of cytokines, oxidative stress, and tissue remodeling enzymes, all of which are recognized facilitators of carcinogenesis [9,10]. Moreover, periodontal pathogens can directly interact with epithelial cells, modulate host immune responses, and disrupt epithelial barrier integrity, thereby promoting genomic instability and malignant transformation [11,12,13]. Among these pathogens, *Porphyromonas gingivalis* has received particular attention due to its ability to invade epithelial cells, inhibit apoptosis, and manipulate signaling pathways involved in cell proliferation and immune evasion [14,15,16].

Epidemiological studies have increasingly reported associations between clinical or radiographic indicators of periodontal disease—such as alveolar bone loss, clinical attachment loss, and gingival recession—and an elevated risk of oral cancer. However, individual studies vary substantially in design, exposure definitions, and confounding control, resulting in heterogeneous and sometimes inconsistent findings [17,18,19]. Consequently, the magnitude and robustness of the association between periodontal disease and OSCC risk remain subjects of debate.

Beyond cancer risk, emerging evidence suggests that periodontal disease and specific periodontal pathogens may also influence cancer prognosis. Chronic oral infection may contribute to tumor progression through persistent inflammation, immune modulation, and direct microbial–tumor interactions. In this context, intratumoral detection of *P. gingivalis* has been associated with more aggressive tumor behavior and poorer survival outcomes in patients with OSCC, although available data are limited and derived from relatively small cohorts [20,21]. A systematic synthesis of this prognostic evidence is therefore warranted.

To date, no comprehensive systematic review and meta-analysis has jointly evaluated the etiological association between periodontal disease and OSCC risk and the prognostic impact of periodontal disease–related factors on survival outcomes in OSCC. Addressing both dimensions within a unified analytical framework is essential to better understand the periodontal–oral cancer axis and its potential clinical implications.

Accordingly, the present systematic review and meta-analysis aimed to: (i) quantitatively assess the association between periodontal disease and the risk of developing oral squamous cell carcinoma (etiological analysis), and (ii) evaluate the prognostic significance of periodontal disease and periodontal pathogens, particularly *Porphyromonas gingivalis*, in relation to survival outcomes among patients with OSCC (prognostic analysis). By integrating epidemiological and prognostic evidence, this study seeks to clarify the role of periodontal disease across the oral cancer continuum, from risk to survival.

## 2. Materials and Methods

### 2.1. Protocol Registration and Reporting Standards

**Figure 1:**
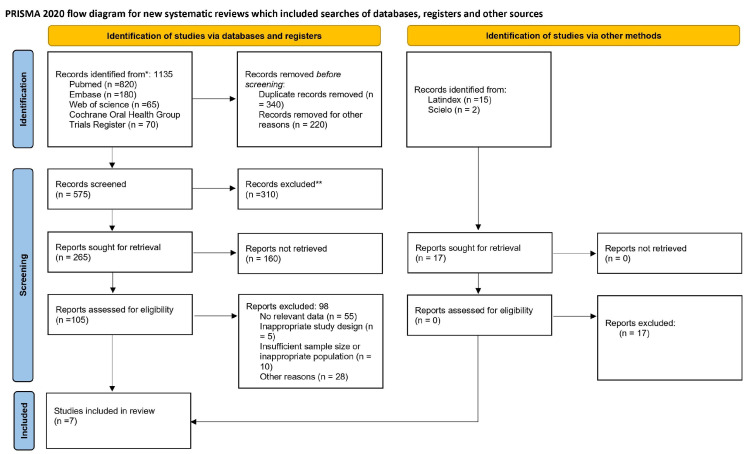
Flowchart of selected studies. * Records identified through database searching and other sources. ** Records excluded after title and abstract screening because they did not meet the predefined inclusion criteria.

The protocol for this systematic review and meta-analysis was prospectively registered in the PROSPERO database (registration number: CRD420261296479) following its initial submission in November 2025. The conduct and reporting of the study adhered to the Preferred Reporting Items for Systematic Reviews and Meta-Analyses (PRISMA) guidelines (Figure 1) [22].

### 2.2. PICO Question

#### 2.2.1. Risk of Oral Squamous Cell Carcinoma (Etiological/Epidemiological Analysis)

In adults aged ≥18 years without a prior diagnosis of oral cancer (P), does exposure to periodontal disease (I)—defined using clinical and/or radiographic criteria such as chronic or aggressive periodontitis, clinical attachment loss, probing depth, or alveolar bone loss—compared with individuals without periodontal disease or with periodontal health (C), associate with an increased risk of developing oral squamous cell carcinoma (O), as reported by observational studies (cohort, case–control, or population-based designs) providing odds ratios, relative risks, or hazard ratios with corresponding 95% confidence intervals?

#### 2.2.2. Survival and Prognosis in Oral Squamous Cell Carcinoma (Clinical–Prognostic Analysis)

In adult patients diagnosed with oral squamous cell carcinoma (P), does the presence of prior or concurrent periodontal disease and/or the detection or burden of periodontal pathogens, particularly *Porphyromonas gingivalis* (I), compared with patients without periodontal disease or without detection of periodontal pathogens (C), associate with worse survival outcomes (O), including overall survival, disease-free survival, and cancer-specific survival, as reported by clinical or observational studies providing hazard ratios with corresponding 95% confidence intervals?

### 2.3. Search Strategy and Database Screening

The identification and screening of eligible studies were conducted using the Rayyan QCRI platform (Qatar Computing Research Institute, Doha, Qatar). A comprehensive and systematic search was performed across multiple electronic databases, including MEDLINE (via PubMed), EMBASE (via OVID), Web of Science, Scopus, the Cochrane Library, ClinicalTrials.gov, and the five World Health Organization (WHO) regional bibliographic databases (AIM, LILACS, IMEMR, IMSEAR, and WPRIM). Conference proceedings were additionally screened through the Conference Proceedings Citation Index.

The search strategy combined controlled vocabulary terms and free-text keywords related to periodontal disease, periodontal clinical parameters, periodontal pathogens, and oral squamous cell carcinoma. Key terms included but were not limited to: “periodontal disease”, “periodontitis”, “clinical attachment loss”, “alveolar bone loss”, “periodontal pathogens”, “*Porphyromonas gingivalis*”, “oral squamous cell carcinoma”, and “oral cancer”. Boolean operators (AND/OR) were applied, and the strategy was tailored to the specific syntax of each database to maximize sensitivity.

To ensure completeness, the electronic search was complemented by a manual screening of reference lists from relevant peer-reviewed articles and reviews in the field. No restrictions were applied regarding publication year during the initial search phase.

### 2.4. Eligibility Criteria

#### 2.4.1. Inclusion Criteria

Observational studies conducted in human adults (≥18 years), including case–control, cohort, or population-based studies, evaluating the association between periodontal disease and oral squamous cell carcinoma (OSCC). Studies assessing periodontal disease using clinical and/or radiographic diagnostic criteria, including periodontitis, clinical attachment loss (CAL), probing pocket depth (PPD), gingival recession, or radiographic alveolar bone loss. Studies in which the diagnosis of oral squamous cell carcinoma was histopathologically confirmed. For the etiological analysis (PICO 1), studies reporting risk estimates for OSCC, including odds ratios (ORs), relative risks (RRs), or hazard ratios (HRs) with corresponding 95% confidence intervals, or providing sufficient data to calculate them. For the prognostic analysis (PICO 2), studies evaluating survival outcomes in patients with OSCC in relation to periodontal disease or periodontal pathogens (e.g., *Porphyromonas gingivalis*), reporting hazard ratios (HRs) with 95% confidence intervals for outcomes such as overall survival (OS), disease-free survival (DFS), or cancer-specific survival (CSS). Studies published in English with full-text availability in peer-reviewed journals.

#### 2.4.2. Exclusion Criteria

Preclinical studies, including in vitro experiments and animal models. Case reports, case series, narrative reviews, systematic reviews, meta-analyses, editorials, letters, and conference abstracts without sufficient quantitative data. Studies not evaluating periodontal disease or periodontal pathogens as the exposure of interest. Studies lacking a clearly defined comparator group, such as individuals without periodontal disease or without pathogen detection. Studies not reporting extractable or estimable effect measures (OR, RR, or HR) with corresponding confidence intervals. Studies evaluating oral cancer outcomes other than OSCC without histologically confirmed OSCC cases or without stratified data specific to OSCC. Duplicate publications or studies using overlapping populations, in which case the most comprehensive or recent dataset was retained. Studies with insufficient methodological information or incomplete outcome reporting that precluded inclusion in quantitative synthesis.

### 2.5. Studies Screening and Data Extraction

An ad hoc data extraction form was developed and independently completed by three reviewers (LCh, AOB, and CGM) to ensure consistency and accuracy. Any discrepancies or uncertainties during data extraction were resolved by consensus with three additional reviewers (BVR, SIML, and CECS) who were blinded to the study hypothesis. For studies evaluating the risk of oral squamous cell carcinoma, the following information was extracted: first author, year of publication, country or region, study design, diagnostic criteria used to define periodontal disease (e.g., clinical attachment loss, probing pocket depth, gingival recession, or radiographic alveolar bone loss), outcome assessed, effect measure (adjusted odds ratio), and main study conclusions (Table 1). For prognostic studies, data extraction included study design and population, assessment of the prognostic factor (e.g., intratumoral *Porphyromonas gingivalis* expression), method used for microbial detection (e.g., immunohistochemical detection in tumor tissue samples), survival outcome, statistical analysis method (Cox proportional hazards model), and principal findings (Table 2). Extracted data were organized into predefined qualitative tables to facilitate systematic comparison and synthesis.

### 2.6. Assessment of Risk of Bias (RoB)

The risk of bias of the included studies was independently assessed by two reviewers (L.Ch and BVR). Any disagreements were resolved through discussion, and when consensus could not be reached, a third reviewer (AOB) acted as adjudicator. For studies evaluating the risk of OSCC associated with periodontal disease (etiological analysis; PICO 1), methodological quality was assessed using the Newcastle–Ottawa Scale (NOS) for case–control studies [27]. This tool evaluates three key domains: selection of study groups, comparability of cases and controls, and ascertainment of exposure, allowing classification of studies as having low, moderate, or high risk of bias. For studies assessing the prognostic impact of periodontal disease or periodontal pathogens on survival outcomes in OSCC (PICO 2), the Quality in Prognosis Studies (QUIPS) tool was applied [28]. This instrument evaluates six domains: study participation, study attrition, prognostic factor measurement, outcome measurement, study confounding, and statistical analysis and reporting. Each domain was rated as low, moderate, or high risk of bias, and an overall judgment was assigned to each study based on domain-level assessments. Particular attention was given to the adequacy of confounding adjustment, especially for tobacco use and alcohol consumption. Risk of bias assessments were incorporated into the interpretation of results and explored through sensitivity analyses.

### 2.7. Certainty of Evidence Assessment

The certainty of the evidence for the main outcomes was evaluated using the Grading of Recommendations Assessment, Development and Evaluation (GRADE) approach. Because the review included observational studies, the certainty of evidence initially started at low and was further evaluated across the following domains: risk of bias, inconsistency, indirectness, imprecision, and publication bias. The certainty of evidence was independently assessed for the two primary outcomes: (1) the association between periodontal disease and the risk of oral squamous cell carcinoma, and (2) the association between intratumoral *Porphyromonas gingivalis* expression and survival outcomes in patients with oral squamous cell carcinoma. The detailed certainty-of-evidence assessment is presented in Supplementary Table S2.

### 2.8. Statistical Analysis

#### 2.8.1. Qualitative Synthesis

A qualitative synthesis of all included studies was first performed to summarize study characteristics and methodological features according to the predefined eligibility criteria (see Data extraction). Studies were categorized into two analytical groups based on the research question:

1. Etiological studies (PICO 1) evaluating the association between periodontal disease and the risk of oral squamous cell carcinoma (OSCC) in non-cancer populations.
2. Prognostic studies (PICO 2) assessing the impact of periodontal disease or periodontal pathogens (e.g., *Porphyromonas gingivalis*) on survival outcomes in patients with OSCC.

Key variables, including study design, periodontal disease definition, outcome measures, effect estimates, and confounding adjustment, were descriptively summarized.

#### 2.8.2. Quantitative Synthesis (Meta-Analysis)

For the etiological analysis (PICO 1), effect estimates reported as odds ratios (ORs), relative risks (RRs), or hazard ratios (HRs) with corresponding 95% confidence intervals (CIs) were extracted or calculated when appropriate. When multiple adjusted models were available, the most fully adjusted estimates were preferentially selected for meta-analysis to minimize residual confounding.

For the prognostic analysis (PICO 2), hazard ratios (HRs) with 95% CIs were extracted for overall survival (OS) and, when available, disease-free survival (DFS) or cancer-specific survival (CSS).

All meta-analyses were conducted using an inverse-variance random-effects model to account for both within-study and between-study variability. A random-effects approach was selected a priori because methodological and clinical heterogeneity across studies was expected, including differences in study design, populations, and definitions of periodontal disease.

Statistical analyses were performed using R software (version 4.3.2; R Foundation for Statistical Computing, Vienna, Austria), employing validated meta-analytic procedures. Between-study heterogeneity was assessed using Cochran’s Q test and quantified with the I^2^ statistic, with values of <25% considered low, 25–50% moderate, and >50% high heterogeneity. The between-study variance (τ^2^) was estimated using the DerSimonian–Laird method.

The robustness of pooled estimates was evaluated through sensitivity analyses using the Hartung–Knapp adjustment for random-effects models, providing more conservative confidence intervals. Statistical significance was defined as a two-sided *p*-value < 0.05. Results were graphically presented using forest plots, reporting pooled effect estimates, 95% CIs, study weights, and reference lines for both the null effect and the overall pooled effect.

### 2.9. Additional Analyses

Leave-one-out sensitivity analyses were performed to assess the influence of individual studies on pooled estimates. Influence diagnostics and outlier detection were conducted using studentized residuals and Cook’s distance to identify potentially influential studies.

For the etiological analysis (PICO 1), Trial Sequential Analysis (TSA) was conducted using O’Brien–Fleming monitoring boundaries to control for random errors due to sparse data and repeated significance testing. A two-sided α of 5% and a statistical power of 80% were applied, assuming an anticipated effect size based on prior epidemiological evidence.

Formal assessment of publication bias using funnel plots or statistical tests was not performed because fewer than ten studies were included in each meta-analysis, a threshold below which such methods are known to be unreliable, in accordance with established methodological recommendations.

## 3. Results

The included studies addressed two distinct research questions. First, etiological studies evaluated the association between periodontal disease and the risk of developing oral squamous cell carcinoma (OSCC) in individuals without cancer. Second, prognostic studies assessed whether periodontal disease–related microbial factors, particularly intratumoral *Porphyromonas gingivalis*, were associated with survival outcomes in patients already diagnosed with OSCC.

### 3.1. Association Between Periodontal Disease and Risk of Oral Squamous Cell Carcinoma

**Figure 2:**
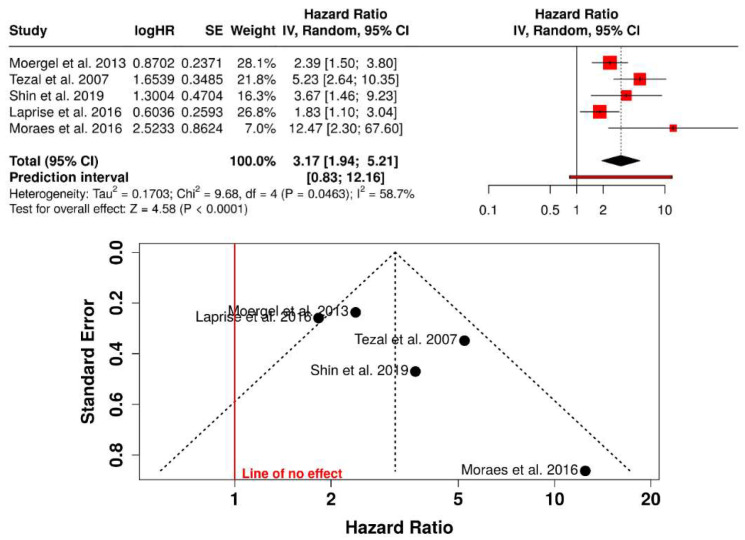
Association between periodontal disease and risk of oral squamous cell carcinoma [17,18,19,23,24].

Five case–control studies involving diverse populations were included in the quantitative synthesis. Periodontal disease was significantly associated with an increased risk of oral squamous cell carcinoma, with a pooled odds ratio (OR) of 3.17 (95% CI: 1.94–5.21; *p* < 0.0001) under a random-effects model. Moderate heterogeneity was observed across studies (I^2^ = 58.7%). The direction of the association was consistent in all included studies. The 95% prediction interval (0.83–12.16) indicated variability in effect magnitude across populations, while maintaining an overall increased risk (Figure 2).

### 3.2. Sensitivity Analyses

**Figure 3:**
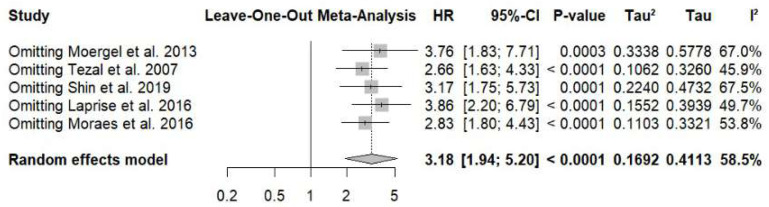
Leave-one-out sensitivity analysis for the association between periodontal disease and risk of oral squamous cell carcinoma [17,18,19,23,24].

Leave-one-out sensitivity analysis demonstrated that the sequential exclusion of individual studies did not materially alter the pooled estimate, which remained statistically significant in all scenarios (OR range: 2.66–3.86). The overall effect size remained stable (OR 3.18; 95% CI: 1.94–5.20), with no evidence that any single study disproportionately influenced the results (Figure 3).

**Figure 4:**
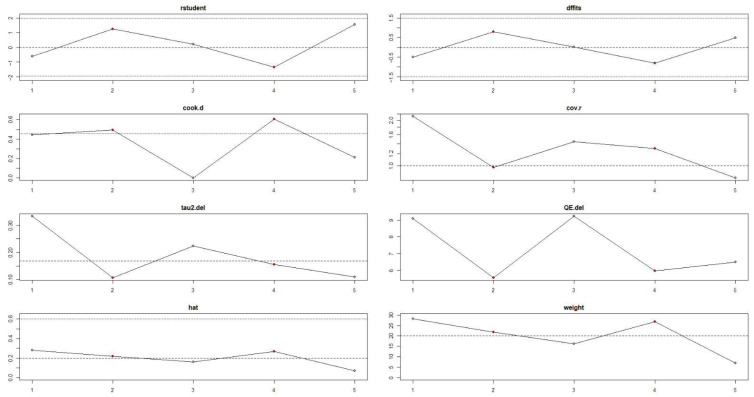
Influence diagnostics for the association between periodontal disease and risk of oral squamous cell carcinoma.

Influence diagnostics did not identify outliers or highly influential studies (|studentized residuals| < 2; Cook’s distance < 4/k). Sequential exclusion of studies produced no substantial changes in the pooled effect or heterogeneity estimates, supporting the stability of the meta-analytic findings (Figure 4).

**Figure 5:**
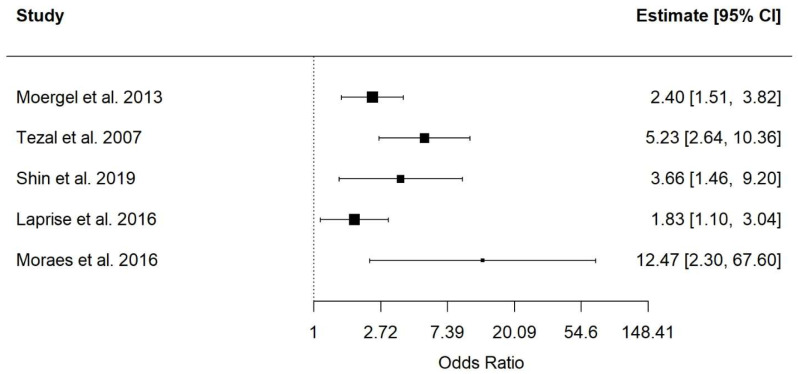
Sensitivity analysis using the Hartung–Knapp adjustment for random-effects models [17,18,19,23,24].

Using the Hartung–Knapp adjustment for random-effects models, the association between periodontal disease and oral squamous cell carcinoma remained statistically significant. The pooled effect estimate was unchanged (OR 3.18), although confidence intervals were wider (95% CI: 1.50–6.72), reflecting a more conservative inference. These results confirm that the observed association was robust and not dependent on the method of variance estimation (Figure 5).

### 3.3. Trial Sequential Analysis

**Figure 6:**
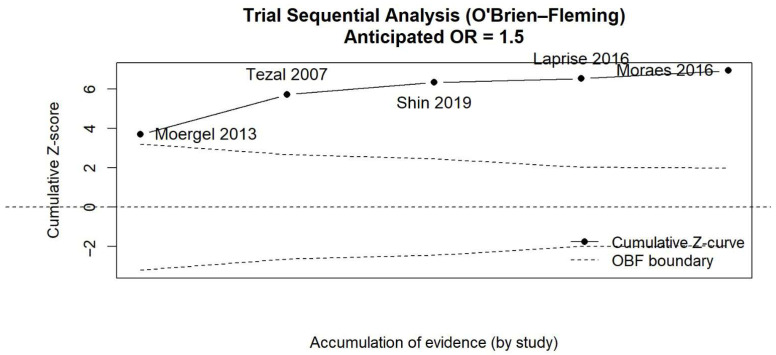
Trial Sequential Analysis of the association between periodontal disease and risk of oral squamous cell carcinoma [17,18,19,23,24].

Trial Sequential Analysis (TSA), applying O’Brien–Fleming monitoring boundaries (anticipated odds ratio = 1.5; two-sided α = 5%; power = 80%), showed that the cumulative Z-curve crossed the trial sequential monitoring boundary from the first included study and remained consistently above this threshold as evidence accumulated. The final Z-value reached 6.94, with an information fraction of approximately 0.98, indicating that the required information size was nearly achieved. These findings suggest that the observed association between periodontal disease and oral squamous cell carcinoma is statistically robust and unlikely to be attributable to random error (Figure 6).

### 3.4. Risk of Bias Assessment of Included Studies

**Figure 7:**
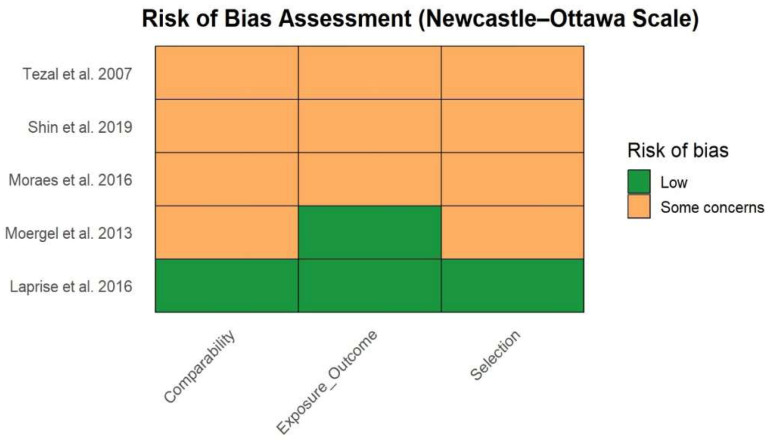
Risk of bias assessment of etiological studies using the Newcastle–Ottawa Scale [17,18,19,23,24].

Risk of bias assessment using the Newcastle–Ottawa Scale indicated an overall low to moderate risk of bias among the included studies. The main methodological concerns were related to comparability and selection domains, primarily due to incomplete adjustment for key confounding factors such as tobacco use and alcohol consumption. No study was judged to have a high risk of bias. Notably, Laprise et al. (2016) [23] demonstrated low risk of bias across all evaluated domains, while the remaining studies presented some concerns in at least one domain, indicating an overall acceptable methodological quality of the evidence (Figure 7).

### 3.5. Association Between Intratumoral Porphyromonas gingivalis and Overall Survival in Oral Squamous Cell Carcinoma

**Figure 8:**
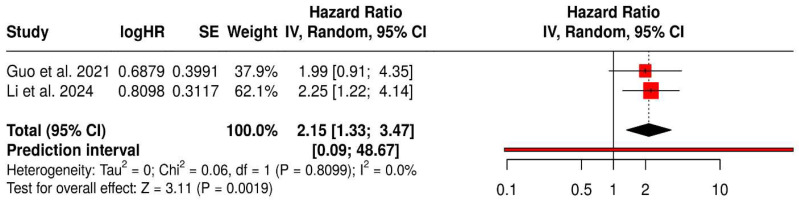
Association between intratumoral *Porphyromonas gingivalis* expression and overall survival in oral squamous cell carcinoma [25,26].

Two cohort studies evaluating intratumoral *Porphyromonas gingivalis* expression as a prognostic factor in patients with oral squamous cell carcinoma were included in the quantitative synthesis. Using an inverse-variance random-effects model, high intratumoral *P. gingivalis* expression was significantly associated with poorer overall survival, with a pooled hazard ratio (HR) of 2.15 (95% CI: 1.33–3.47; *p* = 0.0019). No statistical heterogeneity was observed between studies (I^2^ = 0.0%, τ^2^ = 0), indicating consistent effect estimates across cohorts. Although the 95% prediction interval was wide (0.09–48.67), reflecting the limited number of available studies, the direction and magnitude of the pooled effect support a potential adverse prognostic role of intratumoral *P. gingivalis* in oral squamous cell carcinoma (Figure 8).

### 3.6. Sensitivity Analysis

**Figure 9:**
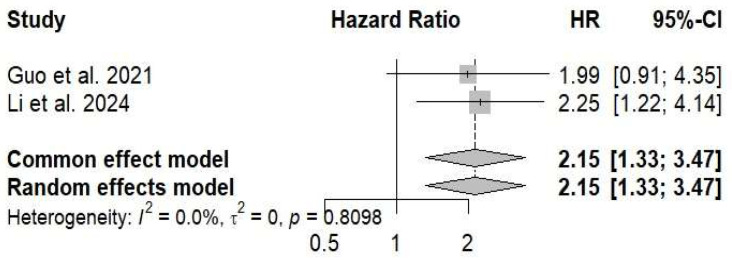
Sensitivity analysis comparing fixed-effect and random-effects models for overall survival in oral squamous cell carcinoma [25,26].

A sensitivity analysis comparing fixed-effect and random-effects models yielded identical pooled estimates for overall survival. The combined hazard ratio remained unchanged under both models (HR 2.15; 95% CI: 1.33–3.47), indicating that the observed association between high intratumoral *P. gingivalis* expression and poorer survival was independent of the statistical model applied. No heterogeneity was detected between studies (I^2^ = 0.0%, τ^2^ = 0; *p* = 0.81), supporting the consistency of the effect across cohorts (Figure 9).

### 3.7. Risk of Bias Assessment

**Figure 10:**
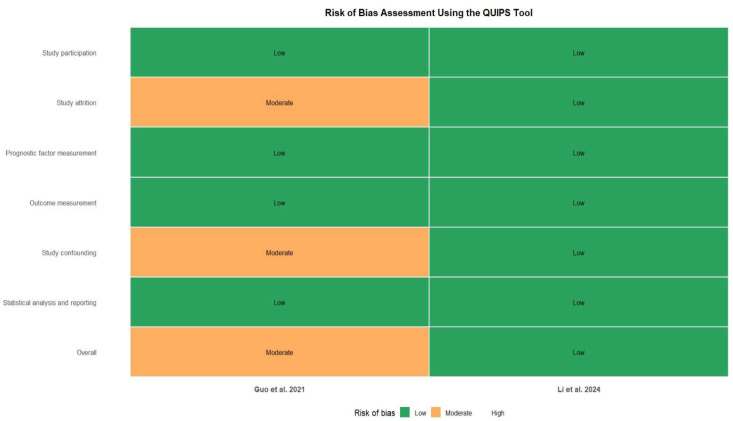
Risk of bias assessment of prognostic studies using the Quality in Prognosis Studies (QUIPS) tool [25,26].

Risk of bias assessment using the QUIPS tool indicated an overall low to moderate risk of bias among the included studies. Most domains were judged to be at low risk, including study participation, prognostic factor measurement, outcome measurement, and statistical analysis. Guo et al. (2021) [25]. presented a moderate risk of bias related to incomplete reporting of attrition and limitations in confounding adjustment, whereas Li et al. (2024) [26] demonstrated a low risk of bias across all evaluated domains. Overall, the methodological quality of the included prognostic evidence was considered acceptable (Figure 10).

## 4. Discussion

The results of this systematic review and meta-analysis indicate that periodontal disease is consistently associated with an increased risk of oral squamous cell carcinoma (OSCC). The observed effect size—approximately a threefold increase in risk—is both clinically meaningful and statistically robust (pooled OR = 3.17; 95% CI: 1.94–5.21), even in the presence of moderate heterogeneity (I^2^ = 58.7%). This between-study variability is not unexpected, given the use of heterogeneous clinical and radiographic definitions to characterize periodontal disease, but it does not alter the uniform direction of the observed association [17,18,19].

Early epidemiological investigations had already suggested a link between chronic periodontal destruction and the development of OSCC. In this context, the study by Tezal et al. provided a key observation by demonstrating that radiographically assessed alveolar bone loss—used as a marker of chronic periodontitis—was independently associated with tongue squamous cell carcinoma, even after rigorous adjustment for smoking and dental variables [17]. Subsequently, Moergel et al. strengthened this evidence by identifying chronic periodontitis as an independent risk factor for OSCC and, notably, suggesting that patients with a history of periodontal treatment exhibited a relatively lower risk, introducing the concept of a potentially modifiable exposure [18].

The external validity of these findings was further reinforced by larger population-based studies. In a general population analysis, Shin et al. demonstrated that radiographic evidence of alveolar bone loss consistent with periodontitis—ranging from mild to severe—was significantly associated with OSCC after comprehensive adjustment for sociodemographic and behavioral factors [19]. Concordantly, the HeNCe Life study conducted in southern India showed that clinical indicators of periodontitis, such as generalized gingival recession, were independently associated with an increased risk of oral cancer, regardless of tobacco, alcohol, and paan consumption [23]. Similar results were reported in a Brazilian hospital-based setting, where Moraes et al. documented a consistent association between the clinical extent of periodontitis and the risk of oral and oropharyngeal cancer [24]. Taken together, these studies suggest that different diagnostic approaches—radiographic or clinical—capture a common underlying chronic inflammatory process that is persistently linked to OSCC.

The consistency observed across primary studies is also reflected in prior quantitative syntheses. Meta-analyses by Yao et al. and by Javed and Warnakulasuriya had already identified a positive association between periodontal disease and oral cancer, although with more conservative estimates, likely due to the more limited availability of adjusted studies at the time [29,30]. More recent meta-analyses, incorporating larger sample sizes and more rigorous control for confounding factors, continue to confirm this relationship across diverse populations [31,32].

A relevant methodological advance is provided by the Mendelian randomization study conducted by Xiao et al., which supported a directional relationship between periodontitis and oral cancer, thereby reducing the likelihood that the observed association is solely attributable to residual confounding or reverse causality [33]. Although this approach does not replace direct clinical evidence, it strengthens the causal plausibility of the reported link.

In parallel, emerging biological evidence supports this association from the perspective of the oral microbiome. Recent systematic reviews have shown that OSCC tumor tissues exhibit a reproducible dysbiotic profile, characterized by a relative enrichment of periodontal pathogens compared with healthy oral tissues [34,35].

At the mechanistic level, several studies have demonstrated that *Porphyromonas gingivalis* can modulate key cellular processes involved in oral carcinogenesis. Notably, Chang et al. described activation of the miR-21/PDCD4/AP-1 pathway as a mechanism through which this pathogen promotes OSCC cell proliferation [36]. Complementary evidence indicates that other periodontal pathogens, such as *Treponema denticola* and *Fusobacterium nucleatum*, contribute to tumor progression through activation of oncogenic signaling pathways and remodeling of the tumor microenvironment [37,38,39]. These observations support the notion that chronic periodontal inflammation and its associated dysbiosis do not merely act as risk markers but rather as active participants in tumor biology.

More recently, attention has shifted toward the potential prognostic impact of periodontal pathogens in established OSCC. Available data indicate that intratumoral presence of *P. gingivalis* is associated with an unfavorable clinical course, with a significant increase in mortality risk (pooled HR = 2.15; 95% CI: 1.33–3.47) and remarkable consistency across studies (I^2^ = 0%) [25,26].

Experimental studies have begun to elucidate the mechanisms underlying this association. It has been shown that outer membrane vesicles derived from *P. gingivalis* can promote tumor migration and invasion through small RNA molecules and disruption of cell adhesion proteins [40]. Additionally, recent work has demonstrated that these vesicles facilitate epithelial–mesenchymal transition and tumor progression by inhibiting ferroptosis via the NF-κB pathway and suppressing innate immune surveillance [41,42]. Complementarily, microbiota-induced activation of γδ T lymphocytes has also been implicated in the promotion of OSCC [43].

From a clinical perspective, these findings reinforce the relevance of periodontal disease as a potentially modifiable factor within the oral cancer risk spectrum. Given its high prevalence, even modest reductions in relative risk could translate into a meaningful population-level impact. Nevertheless, interpretation of these results should remain cautious, considering the predominance of case–control designs and the still limited number of available prognostic studies [17,19,24].

Furthermore, accumulating evidence suggests that chronic inflammation and immune dysregulation play an important role in the pathogenesis and progression of oral squamous cell carcinoma. Periodontal pathogens and tumor-associated microbial dysbiosis may promote carcinogenesis through persistent inflammatory signaling, modulation of the tumor microenvironment, and alterations in host immune responses. In this context, systemic and local inflammatory markers have been associated with disease progression and prognosis in patients with OSCC, further supporting the biological plausibility of the link between periodontal disease, microbial factors, and oral carcinogenesis [44].

Despite this biological plausibility, several methodological considerations must be taken into account when interpreting the present findings. First, the evidence base is derived primarily from observational studies, particularly case–control designs, which are inherently susceptible to confounding and selection bias. Periodontal disease shares several major determinants with oral squamous cell carcinoma, including tobacco use, alcohol consumption, socioeconomic disadvantage, and potentially human papillomavirus (HPV) exposure. Although many of the included studies adjusted for some of these behavioral risk factors, the extent and consistency of confounder control varied across studies, and residual confounding cannot be excluded. Consequently, the observed association between periodontal disease and OSCC should be interpreted as an epidemiological correlation rather than definitive evidence of causality.

Second, heterogeneity in exposure assessment represents an additional limitation. The included studies did not use a single standardized definition of periodontal disease; instead, different clinical or radiographic indicators were employed, including alveolar bone loss, clinical attachment loss, probing pocket depth, and gingival recession. These measures capture related but not identical aspects of periodontal destruction and may differ in sensitivity, chronicity, and susceptibility to measurement error. Such variability likely contributed to the moderate heterogeneity observed in the etiological meta-analysis.

Third, although moderate heterogeneity was detected, the small number of included studies limited the feasibility of performing subgroup analyses or meta-regression to further explore potential sources of variability. Methodological guidelines generally discourage these analyses when fewer than ten studies are available, as they may produce unstable or misleading estimates. Future meta-analyses, including a larger number of studies, may allow a more detailed exploration of heterogeneity related to disease severity, tumor subsite, geographic population, or adjustment for behavioral risk factors.

An additional limitation concerns the prognostic meta-analysis, which was based on only two cohort studies evaluating intratumoral *Porphyromonas gingivalis*. Although both studies assessed the same microbial factor and reported hazard ratios for overall survival using comparable statistical approaches, the small number of studies limits the robustness and generalizability of the pooled estimate. Furthermore, potential methodological differences in microbial detection methods, threshold definitions for high expression, and analytical adjustment strategies may have affected comparability between studies. For these reasons, the prognostic findings should be interpreted as exploratory and hypothesis-generating rather than definitive evidence.

Taken together, these methodological limitations do not negate the biological plausibility of the periodontal–oral cancer link but underscore the need for cautious interpretation. Larger prospective studies using standardized periodontal definitions, rigorous confounder adjustment, and harmonized microbial detection approaches will be essential to clarify the causal and prognostic role of periodontal disease and periodontal pathogens in oral squamous cell carcinoma.

## 5. Conclusions

This systematic review and meta-analysis provide evidence that periodontal disease is significantly associated with an increased risk of oral squamous cell carcinoma and that elevated intratumoral expression of *Porphyromonas gingivalis* is associated with poorer overall survival. Together, these findings support a contributory role of chronic periodontal inflammation and periodontal pathogens across the oral cancer continuum, encompassing both carcinogenesis and disease progression. Although prognostic evidence remains limited, the consistency of the observed associations suggests that periodontal disease and *P. gingivalis* may represent clinically relevant and potentially modifiable risk and prognostic factors, underscoring the need for well-designed prospective and mechanistic studies to clarify causality and therapeutic implications.

## Figures and Tables

**Table 1:** Characteristics of case–control studies evaluating the association between periodontal disease and the risk of oral squamous cell carcinoma.

Author (Year)	Country/ Region	Study Design	Sample Size	Diagnostic Criteria for Periodontal Disease.	Outcome	Measure	Main Study Conclusion
Tezal et al., 2007 [17]	USA	CC	51 cases/54 controls (*n* = 105)	Radiographic alveolar bone loss (mm)	OSCC (tongue)	aOR	Alveolar bone loss was independently associated with an increased risk of tongue squamous cell carcinoma, even after adjustment for smoking and dental factors.
Moergel et al., 2013 [18]	Germany	CC (retrospective)	178 cases/123 controls (*n* = 301)	Mean radiographic alveolar bone loss	OSCC	aOR	Chronic periodontitis, assessed by alveolar bone loss, was identified as an independent risk factor for OSCC; periodontal treatment showed a potential protective effect.
Shin et al., 2019 [19]	South Korea	Population-based CC	146 cases/278 controls (*n* = 424)	Radiographic alveolar bone loss (mild–severe)	OSCC	aOR	The presence of radiographic periodontitis was significantly associated with an increased risk of OSCC after extensive adjustment for sociodemographic, behavioral, and metabolic factors.
Laprise et al., 2016 [23]	India	Population-based CC	306 cases/328 controls (*n* = 634)	Generalized gingival recession (history of periodontitis)	OC	aOR	Clinical indicators of chronic periodontitis were associated with a significantly increased risk of oral cancer, independent of tobacco, alcohol, and paan consumption.
Moraes et al., 2016 [24]	Brazil	Hospital-based CC	306 cases/328 controls (*n* = 634)	Chronic periodontitis (clinical extent: CAL/PPD)	OSCC/OPSCC	aOR	The extent of chronic periodontitis showed a strong association with oral and oropharyngeal cancer, even after adjustment for smoking and alcohol consumption.

Abbreviations: CC: Case–control, OSCC: Oral Squamous Cell Carcinoma, OPSCC: Oropharyngeal Squamous Cell Carcinoma, OC: Oral Cancer, aOR: Adjusted Odds Ratio, CAL: Clinical Attachment Level, PPD: Probing Pocket Depth.

**Table 2:** Qualitative characteristics and risk of bias of prognostic studies evaluating intratumoral *Porphyromonas gingivalis* in oral squamous cell carcinoma.

Study	Design/Population	Prognostic Factor	Sample Size	Outcome	Statistical Analysis	Main Finding
Guo et al., 2021 [25]	Retrospective cohort; patients with OSCC	Intratumoral *P. gingivalis* expression (IHC; strong vs. weak)	205 OSCC patients	Overall survival	Cox proportional hazards model (multivariable)	High *P. gingivalis* expression was associated with poorer survival, although the confidence interval included the null
Li et al., 2024 [26]	Retrospective cohort; patients with OSCC	Intratumoral *P. gingivalis* expression (IHC; strong vs. weak)	205 OSCC patients	Overall survival	Cox proportional hazards model (multivariable)	High *P. gingivalis* expression was an independent predictor of worse overall survival

Abbreviations: OSCC: oral squamous cell carcinoma; IHC: immunohistochemistry.

## Data Availability

The raw data supporting the conclusions of this article will be made available by the authors, without undue reservation.

## Supplementary Materials

The following supporting information can be downloaded at https://www.mdpi.com/article/10.3390/jcm15083161/s1: Table S1: PRISMA_2020_checklist. Table S2: Certainty of evidence assessment using the GRADE approach for the main outcomes.
