# Supplementary material for: Periodontal Disease and Oral Squamous Cell Carcinoma: A Systematic Review and Meta-Analysis of Risk and Survival Outcomes
Source: J Clin Med. 2026 Apr 21;15(8):3161. doi: 10.3390/jcm15083161 (PMC13117503; doi:10.3390/jcm15083161)
Supplement: Supplementary file 1 [file jcm-15-03161-s001.zip › jcm-4171596-supplementary.pdf]

Supplementary Table S2. Certainty of evidence assessment using the GRADE approach for the main outcomes.

Outcome	No. of studies	Study design	Risk of bias	Inconsistency	Indirectness	Imprecision	Publication bias	Overall certainty of evidence (GRADE)	Explanation
Association between periodontal disease and risk of oral squamous cell carcinoma (OSCC)	5	Observational (case–control)	Serious	Serious (moderate heterogeneity, I^2 = 58.7%)	Not serious	Not serious	Not assessable (<10 studies)	**Low**	Evidence downgraded due to observational design and potential residual confounding (e.g., smoking, alcohol, socioeconomic factors). Moderate heterogeneity was observed among studies, although the direction of effect was consistent.
Association between intratumoral *Porphyromonas gingivalis* expression and overall survival in OSCC	2	Observational (cohort)	Serious	Not serious (I^2 = 0%)	Not serious	Serious (limited number of studies and wide prediction interval)	Not assessable	**Very Low**	Evidence downgraded due to observational design and serious imprecision resulting from the very small number of studies and limited sample size in prognostic analysis.
